# Fine mapping of a panicle blast resistance gene *Pb-bd1* in *Japonica* landrace Bodao and its application in rice breeding

**DOI:** 10.1186/s12284-019-0275-0

**Published:** 2019-03-25

**Authors:** Nengyan Fang, Xiaorui Wei, Lingtong Shen, Yao Yu, Mengya Li, Congfei Yin, Wanwan He, Changhong Guan, Hao Chen, Hongsheng Zhang, Yongmei Bao

**Affiliations:** 1 State Key Laboratory of Crop Genetics and Germplasm Enhancement, Jiangsu Collaborative Innovation Center for Modern Crop Production, Cyrus Tang Innovation Center for Seed Industry, College of Agriculture, Nanjing Agricultural University, Nanjing, 210095 China; 2 Institute of Crop Science, Fujian Academy of Agricultural Science, Fuzhou, 350013 China; 3 Xuzhou Academy of Agricultural Science, Xuzhou, 221131 China

**Keywords:** Rice, Panicle blast resistance, QTL, Fine mapping, MAS, Introgression lines, Resistance breeding

## Abstract

**Background:**

Rice blast caused by *Magnaporthe oryzae* is the most devastating disease in rice production. Compared with seedling blast, panicle blast is considered to be more destructive, which can occur without being preceded by severe seedling blast. However, panicle blast resistance research is rarely reported.

**Results:**

Bodao, a *japonica* landrace from Taihu Lake region, showed a high level of panicle blast resistance. In this study, a mapping population of 212 recombination inbreeding lines (RILs) was developed from a cross of Bodao and the susceptible cultivar Suyunuo, and the RILs were evaluated for panicle blast resistance in three trials. Two quantitative trait loci (QTLs) *qPb11–1* and *qPb6–1* for panicle-blast resistance were identified, including a major QTL *qPb11–1* (*Pb-bd1*) on chromosome 11 of Bodao explaining from 55.31% to 71.68% of the phenotype variance, and a minor QTL *qPb6–1* on chromosome 6 of Suyunuo explaining from 3.54% to 6.98% of the phenotype variance. With the various segregation populations, *Pb-bd1* was fine mapped in a 40.6 Kb region flanked by markers BS83 and BS98, and six candidate genes were identified within this region, including one gene encoding NAC domain-containing protein, one gene encoding unknown expression proteins, two genes encoding nucleotide binding site-leucine rich repeat (NBS-LRR) type disease resistance proteins, and two genes encoding von Willebrand factor type A (VWA) domain containing proteins. For application in rice breeding, three introgression lines of *Pb-bd1*with significantly enhanced panicle blast resistance were developed by using molecular assisted method (MAS) from the commercial variety Nanjing46 (NJ46).

**Conclusion:**

Two QTLs, *qPb11–1*(*Pb-bd1*) and *qPb6–1* conferring panicle blast resistance, were identified from *japonica* landrace Bodao and Suyunuo.*qPb11–1*(*Pb-bd1*) was fine mapped in a 40.6 Kb region flanked by marker BS83 and BS98. Three introgression lines of *Pb-bd1*with significantly enhanced panicle blast resistance were developed by MAS method from the commercial variety NJ46. It indicated that *Pb-bd1* would be useful gene source in panicle blast resistance breeding.

**Electronic supplementary material:**

The online version of this article (10.1186/s12284-019-0275-0) contains supplementary material, which is available to authorized users.

## Background

Rice (*Oryza sativa* L.) is one of the major food crops, and it is the staple food for more than half of the world population (Sharma et al. 2012). Rice blast, caused by the fungal pathogen *Magnaporthe oryzae*, is one of the most devastating diseases worldwide, which can occur throughout entire rice growth period (Ou 1985). Up to date, more than 100 blast resistance genes were identified in different genotypes of rice and 28 resistance genes among them have been cloned (Ashkani et al. 2016). *Pi-b* was the first cloned blast resistance gene through a map-based cloning strategy (Wang et al. 1999). Four genes *Pi2*, *Pi9*, *Pi-gm* and *Piz-t* were located in *Pi2* cluster on chromosome 6 (Zhou et al. 2006; Deng et al. 2006; Qu et al. 2006). Six genes *Pik*, *Pik-h*, *Pik-m*, *Pik-p*, *Pi1* and *Pi-ke* were located in *Pik* cluster on chromosome 11 (Ashikawa et al. 2008; Chen et al. 2015; Hua et al. 2012; Sharma et al. 2005; Yuan et al. 2011; Zhai et al. 2011). The majority of cloned resistance genes encoded nucleotide binding site-leucine rich repeat (NBS-LRR) proteins (Chen et al. 2010), except for *Pi-d2* (encoding a B-lectin receptor kinase) (Kouzai et al. 2013), recessive gene *pi21* (encoding a proline-rich protein) (Fukuoka et al. 2009), *Bsr-d1* (encoding a C2H2-type transcription factor protein) (Li et al. 2017), and *Bsr-k1* (encoding a tetratricopeptide repeats-containing protein) (Zhou et al. 2018). Deng et al. (2017) revealed that epigenetic regulation of *Pi-gmR* and *Pi-gmS* can balance the blast resistance and yield in rice. *Pi-gmR* confered broad-spectrum resistance, and *Pi-gmS* can increase rice production to counteract the yield lost caused by *Pi-gmR*. Li et al. (2017) reported *Bsr-d1* was an C_2_H_2_-type transcription factor conferring broad-spectrum blast resistance, and low expression of this gene could enhance disease resistance by inhibiting degradation of H_2_O_2_.

Among the 28 cloned resistance genes, only *Pb1* was a panicle blast resistance gene. *Pb1* was isolated from the *indica* cultivar Modan, and encoded a coiled coil-nucleotide binding site-leucine rich repeat (CC-NBS-LRR) protein, conferring durable and broad-spectrum resistance to rice blast (Hayashi et al. 2010; Inoue et al. 2013). *Pi25* and *Pi64* were associated with both seedling blast and panicle blast resistance and encoded CC-NBS-LRR proteins (Wu et al. 2005; Chen et al. 2011; Ma et al. 2015). In total, eight panicle blast resistant QTLs have been identified. Ishihara et al. (2014) identified two panicle blast resistance QTLs *qPbm11* and *qPbm9* on chromosome 11 and 9 in *japonica* cultivar Miyazakimochi. Fang et al. (2016) found one major panicle blast resistance QTL *qPbh-11-1* and one minor QTL *qPbh-7-1* from *japonica* landrace Heikezijing. Wang et al. (2016) identified one major panicle blast resistance QTL *qPbj-11-1* and three minor QTLs *qPbj-7-1*, *qPbj-6-1* and *qPbj-9-1* from *japonica* landrace Jiangnanwan.

Compared with seedling blast, panicle blast is considered to be more destructive, which can occur without being preceded by severe seedling blast (Katsube and Koshimizu 1970; Hwang et al. 1987; Zhu et al. 2005). Panicle blast can cause direct yield losses up to 70% even 100% in fields by affecting grain sterility, rotting the branch and neck, even losing the entire panicle (Liu et al. 2014; Khan et al. 2014; Roumen 1992; Bonman et al. 1991; Chin 1975; Lu et al. 2015). However, there are few reports about resistance genes or QTLs of panicle blast and the correlation between seedling blast and panicle blast (Koh et al. 1987; Zhuang et al. 2002; Bonman 1992; Fang et al. 2016; Sirithunya et al. 2002). The major difficulty for panicle blast researching is that the inoculation and phenotype identification should be conducted in the fields (Liu et al. 2016; Sirithunya et al. 2002; Zhuang et al. 2002).

So far, the most economical and effective way to control blast disease is introducing resistance genes into susceptible elite cultivars (Hulbert et al. 2001). The resistance genes as *Pi1*, *Pi5*, *Piz-5*, *Pita* and *Pi-gm* have been introgressed into various elite cultivars by marker-assisted selection (MAS) method (Sharma et al. 2012; Deng et al. 2017). However, few genes were applied for controlling the panicle blast resistance. Bodao is a *japonica* landrace from Taihu Lake region and exhibited high leaf blast resistance (Li et al. 2007a, 2007b; Huan et al. 2014). In this study, we fine mapped a panicle blast resistance gene *Pb-bd1* in *japonica* landrace Bodao, and introduced *Pb-bd1* into commercial *japonica* cultivar Nanjing 46 (NJ46) for panicle blast resistance breeding.

## Methods

### Plant materials and growth

Bodao, a *japonica* rice (*Oryza sativa* L.) landrace from Taihu Lake region in China, showed broad-spectrum resistance to rice blast (Li et al. 2007b). Suyunuo, another *japonica* landrace from the same region, was susceptible to the blast. A RIL population (F_2:7_) consisting of 212 lines, derived from the cross of Bodao and Suyunuo, was used for QTL mapping of panicle blast resistance in this study.

The RIL population and their parents were grown in the Jiangpu experiment station (Nanjing, Jiangsu Province) in 2014 and 2015 and in the Lingshui experiment station (Lingshui, Hainan Province) in 2014 respectively. Twenty plants of each RIL were grown in two rows per plot. Bodao and Suyunuo were grown adjacent to the plots, as resistant and susceptible controls, respectively. At the booting stage panicles of these plants were inoculated with the blast pathogen.

### Inoculation and resistance evaluation

The strain Hoku1 of blast pathogen (*Magniporthe oryzae*) was used for inoculation in this study, which was provided by Institute of Crop Science, Chinese Academy of Agricultural Sciences. 212 RILs and two parents were inoculated with the pathogen by an injection method as described by Liu et al. (2007). Three panicles of per plant and five plants for each line were inoculated with conidial suspension (3 × 10^4^ conidia/ml). Diseased grain rates were evaluated based on visual assessment of disease severity 3 weeks after inoculation as described by Asaga (1981).

### Molecular marker development

According to the International Rice Microsatellite Initiative (IRMI, http://www.gramene.org), 2257 SSR markers were adopted for polymorphism analysis between Bodao and Suyunuo. To find putative InDels, sequence alignments were performed between *japonica* Nipponbare (http://rgp.dna.affrc.go.jp/) and *indica* 93–11 (http://www.gramene.org). InDel markers were designed by Primer Premier 5.0, and identified with 8% polyacrylamide gel electrophoresis (PAGE).

### Genetic map construction and QTL mapping

With the genotypes and panicle blast resistance phenotypes of all 212 RILs, linkage map construction and QTLs mapping were carried out by the software ICIMapping 4.01 (http://www.isbreeding.net/software). The software parameters were set as follows: a LOD threshold of 2.5, walking speed of 1.0 cM, and calculated from 1000 permutation at a probability of 0.01.

### Fine mapping of *Pb-bd1* and prediction of candidate genes

BC_1_F_1,_ BC_2_F_1,_ BC_3_F_1_, BC_4_F_1_ and BC_5_F_1_ plants were obtained from a cross between Bodao and Suyunuo and backcrossed with recurrent parents Suyunuo. BC_4_F_3_ and BC_5_F_3_ populations were obtained from selfing of heterozygous BC_4_F_2_ and BC_5_F_2_, respectively. In this study, 3632 BC_3_F_2_, 5240 BC_4_F_3_, 1200 BC_5_F_2_ and 2928 BC_5_F_3_ plants were used for fine mapping the target QTL *Pb-bd1*. The primers of PCR-based markers used for fine mapping *Pb-bd1* are shown in Additional file 1: Table S1.

The genomic sequences in the region of markers BS83 and BS98 on chromosome 11 were downloaded from the RGP (Rice Genome research Program) Web site (https://rgp.dna.affrc.go.jp/index.html.en). Open reading frames in the target region of *Pb-bd1* were predicted by GENSCAN (http://genes.mit.edu), FGENSH (http://linux1.softberry.com/), and Rice Genome Automated Annotation System (RiceGAAS) (http://rgp.dna.affrc.go.jp) software (Sakata et al. 2002).

### Expression analysis of candidate genes

The GENEVESTIGATOR (http://genevestigator.com/gv/) and Rice Oligo Array Database (http://ricearray.org) were employed to analyze the expression of candidate genes based on 1154 Affymetrix microarray datasets (http://www.ricearray.org/). The expression patterns of five candidate genes *P1*, *P3-P6* were detected in various rice tissues and blast-fungi inoculated seedlings by GENEVESTIGATOR.

The immature panicles at breaking stage of Bodao and Suyunuo inoculated by blast strain Hoku1, were collected at 2 h, 4 h, 8 h, 12 h, 24 h, 48 h and 72 h after inoculation for the expression analysis of candidate genes. The expressions of six candidate genes were detected by real-time PCR methods as described by Huang et al. (2008). The fold changes of target candidate genes relative to the reference gene (18 s-rRNA) was calculated by the 2^-△△CT^ method (Livak and Schmittgen, 2001). All reactions were performed in three replicates. The primers for 6 candidate genes and 18 s-rRNA in quantitative real-time PCR assay are shown in Additional file 2: Table S2.

### Development of ingression lines with *Pb-bd1*

As a blast-resistance donor, Bodao was crossed with a commercial *japonica* cultivar Nanjing 46 (NJ46) in 2012 at Lingshui. NJ46 was used as a recurrent parent, the BC_1_F_1_ and BC_2_F_1_ plants with panicle-blast resistance phenotype were selected after inoculated with Hoku1 at Nanjing in 2013. Based on the QTL mapping results at Nanjing in 2014, the panicle blast resistant plants of BC_3_F_1_, BC_3_F_2_, BC_3_F_3_, and BC_3_F_4_ were selected with *Pb-bd1* linked markers RM7654 and BS79, and the selection was also combined with panicle blast inoculation and agronomy traits identification in the fields.

Three introgression lines (NJ46 + *Pb-bd1*(a), NJ46 + *Pb-bd1*(b), NJ46 + *Pb-bd1*(c)) carrying *Pb-bd1* gene, were grown at Nanjing in 2015 for evaluating their panicle blast resistance and agronomic characters, including the diseased grains rates, plant height, grain number/panicle, 1000-grain weight. Diseased grain rates of three introgression lines were evaluated with an injection method as described as above. The introgression lines were also grown in the natural disease-nurseries in Changsha, Hunan province and in Jintan and Ganyu, Jiangsu province for panicle blast evaluation. Ten plants each row and three rows of each introgression line were grown for the evaluation of panicle blast resistance in the natural disease-nurseries. The panicle blast score (1–9) was identified as describe as Ahn (1994).

## Results

### Characterization of resistance to panicle blast in Bodao

**Fig. 1:**
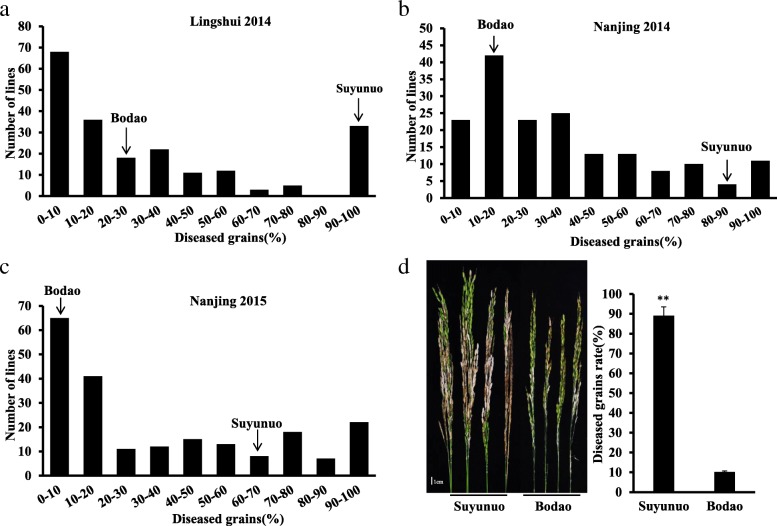
Characterization of panicle blast severity distribution in 212 F_2:7_ RILs. **a**-**c** Distribution of panicle blast diseased grain rate (%) of RILs at Lingshui and Nanjing in 2014, and Nanjing in 2015, respectively. **d** Panicle blast resistant phenotype of Bodao and Suyunuo after inoculation with strain Hoku1 for 3 weeks

Two parents Bodao and Suyunuo, and 212 RILs derived from these two parents were inoculated with blast strain Hoku1 at the booting stage for panicle blast evaluation at Nanjing and Lingshui in 2014, and Nanjing in 2015. The results from three trials showed that Bodao was high resistant to panicle blast with 9.90–25.92% of diseased grains, while Suyunuo susceptible with 63.15–100.00% of diseased grains (Table 1). The frequency distributions of 212 RILs with various diseased grains rates for three trials were asymmetric and continuous, and the distributions were all predisposed resistance-inclined distribution (Fig. 1). In Pearson’s correlation analysis, the resistance to panicle blast each RIL was a significant correlation in various trails (*P* ≤ 0.01) (Table 2).

**Table 1:** Phenotypic values of panicle blast resistance to isolate Hoku1 in F_2:7_ RIL population

Location and year	Isolate	Parents		RIL population^b^					
		Bodao	Suyunuo	Mean	Max	Min	SD^c^	Skewness	Kurtosis
Lingshui2014	Hoku1	25.92 ± 3.77%^a^ (R)	100% (S)	33.85%	100%	0	0.3357	1.0163	−0.3540
Nanjing 2014		10.19 ± 3.41% (R)	89.08 ± 5.90% (S)	35.55%	100%	0	0.2674	0.8126	−0.2598
Nanjing 2015		9.90 ± 1.73% (R)	63.15 ± 9.36% (S)	35.80%	100%	0	0.3236	0.6711	−0.9655

^a^means diseased grains (%);

^b^RIL sample size *n* = 212, replications r = 3;

^c^standard deviation

**Table 2:** Correlation analysis of panicle blast resistance of F_2:7_ RILs in three trails

	Lingshui 2014	Nanjing 2014	Nanjing 2015
Lingshui 2014	1		
Nanjing 2014	0.685**	1	
Nanjing 2015	0.803**	0.792**	1

n = 212, “**” means P ≤ 0.01

### QTL identification of panicle blast resistance in Bodao

**Fig. 2:**
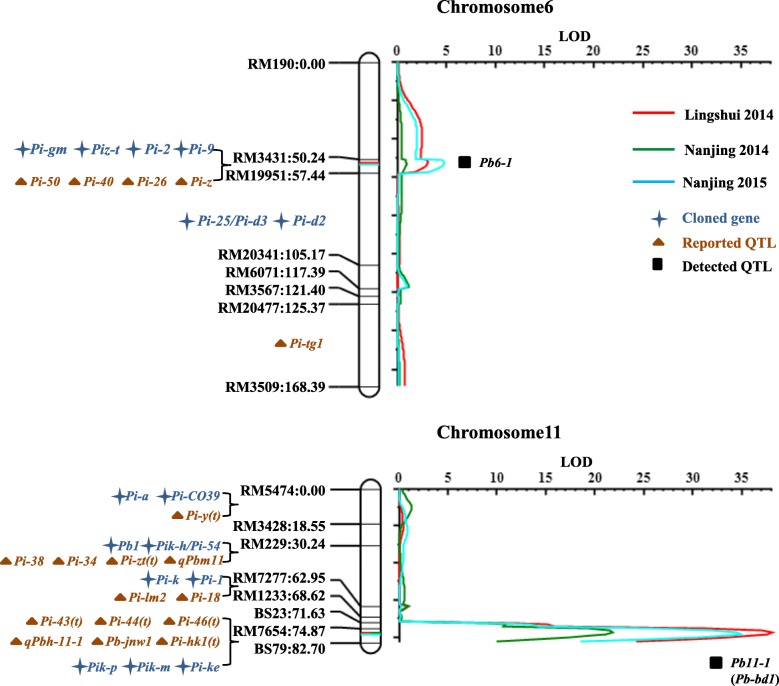
Identification of panicle blast resistance QTLs in Bodao by QTL mapping method. Marker names and their positions are shown on the left linkage group. The color lines indicate logarithm of the odds (LOD) scores

A genetic map with total 1303.34 cM and average 14.98 cM between two adjacent SSR markers was constructed with 87 polymorphic SSR markers selected from 2257 SSR markers. Two panicle blast resistance QTLs *qPb11–1* and *qPb6–1* were identified by inclusive composite interval mapping (ICIM) method with phenotypic data from three trials at Nanjing and Lingshui in 2014, and Nanjing in 2015 (Table 3, Fig. 2). A major QTL *qPb11–1* was detected between marker RM7654 and BS79 on chromosome 11 in all three trials, designated as *Pb-bd1* (Fig. 2). It could explain 64.10%, 56.30% and 73.00% of phenotypic variance with LOD scores of 37.63, 21.58 and 35.71, respectively. Another minor QTL *qPb6–1* was detected between marker RM3431 and RM19951 on chromosome 6 in two trials at Lingshui in 2014 and Nanjing in 2015, explaining 4.97% and 6.06% of phenotypic variance with LOD scores of 4.24 and 4.28, respectively (Fig. 2).

**Table 3:** Identification of panicle blast resistance QTLs in F_2:7_ RILs population

QTL	Location and year	Chromosome	Region	LOD	PVE(%)	Add
*qPb6–1*	Lingshui 2014	6	RM3431-RM19951	4.2383	4.9675	0.0747
	Nanjing 2015	6	RM3431-RM19951	4.2843	6.0634	0.0796
*qPb11–1(Pb-bd1)*	Lingshui 2014	11	RM7654-BS79	37.6284	64.0961	−0.2872
	Nanjing 2014	11	RM7654-BS79	21.578	56.3005	−0.2143
	Nanjing 2015	11	RM7654-BS79	35.7131	73.0038	−0.2889

### Fine mapping of *Pb-bd1*

**Fig. 3:**
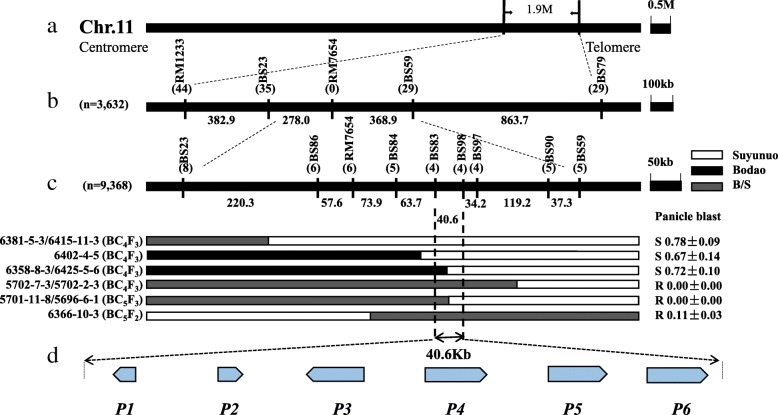
Fine mapping of *Pb-bd1*in Bodao. **a** *Pb-bd1* was located between RM1233-BS79. **b** Seventy-three recombinants were screened from 3632 BC_3_F_2_, and *Pb-bd1* was located between markers BS23 and BS59. **c** 13 recombinants were screened from 5240 BC_4_F_3_, 1200 BC5F2 and 2928 BC_5_F_3_ population, and *Pb-bd1* was finally flanked by markers BS83 and BS98 in the region of 40.6 Kb. **d** Six *Pb-bd1* candidate genes were predicted and the arrows represent the direction of genes.

The *Pb-bd1* gene was mapped between SSR markers RM1233 and BS79 on chromosome 11 using 11 resistant recombinants screened from 45 BC_1_F_1_ plants (Fig. 3a). With segregated population of 3632 BC_3_F_2_, 44, 35, 29 and 29 recombinants were identified by the markers RM1233, BS23, BS59, and BS79, respectively. The results showed that *Pb-bd1* could be mapped in the region of markers BS23 and BS59 (Fig. 3b). Thirteen recombinants were obtained by further screening segregated populations of 5240 BC_4_F_3_, 1200 BC_5_F_2_ and 2928 BC_5_F_3_, including 8, 6, 6, 5, 4, 4, 4, 5 and 5 recombinants identified by markers BS23, BS86, RM7654, BS84, BS83, BS98, BS97, BS90 and BS59, respectively (Fig. 3c). These 13 recombinants were inoculated with blast strain Hoku1 to evaluate their panicle blast resistance phenotypes. The *Pb-bd1* was finally narrowed in the 40.6 kb region between markers BS83 and BS98.

### Candidate genes predicted and their expression

The 40.6 kb of target region conferring *Pb-bd1* in the *japonica* Nipponbare sequence (27,803,976-27,844,340) was covered by two BAC clones OSJNBa0085H07 and OSJNBb0049B20. Six candidate genes were predicted in this region (Fig. 3d), including *P1*, *LOC_Os11g45950*; *P2*, *LOC_Os11g45960*; *P3*, *LOC_Os11g45970*; *P4*, *LOC_Os11g45980*; *P5*, *LOC_Os11g45990*; *P6*, *LOC_Os11g46000*. Among these candidate genes, *P1* encodes a NAC domain-containing protein, P2 encodes an unknown expressed protein, *P3* and *P4* encode NBS-LRR type disease resistance proteins, and *P5* and *P6* encode von Willebrand factor type A domain (VWA) containing proteins. Compared the genomic sequences of these six genes between two parents Bodao and Suyunuo, *P1*, *P4*, *P5* and *P6* showed differences, and *P3* no difference (Table 4), while *P2* only existed in resistant parent Bodao.

**Table 4:** The predicted candidate genes at the *Pb-bd1* region in Bodao

No.	Annotated genes	position	protein length (AA)	Prediction function	Sequence difference between two parents
*P1*	LOC_Os11g45950	27,804,967–27,804,177	137	NAC domain-containing protein 90, putative, expressed	Yes
*P2*	LOC_Os11g45960	27,806,906–27,807,244	113	expressed protein	Yes
*P3*	LOC_Os11g45970	27,818,431–27,812,251	1024	NBS-LRR type disease resistance protein, putative, expressed	No
*P4*	LOC_Os11g45980	27,820,309–27,824,920	853	NBS-LRR type disease resistance protein, putative, expressed	Yes
*P5*	LOC_Os11g45990	27,828,621–27,835,246	634	von Willebrand factor type A domain containing protein, putative, expressed	Yes
*P6*	LOC_Os11g46000	27,841,513–27,845,382	599	von Willebrand factor type A domain containing protein, putative, expressed	Yes

**Fig. 4:**
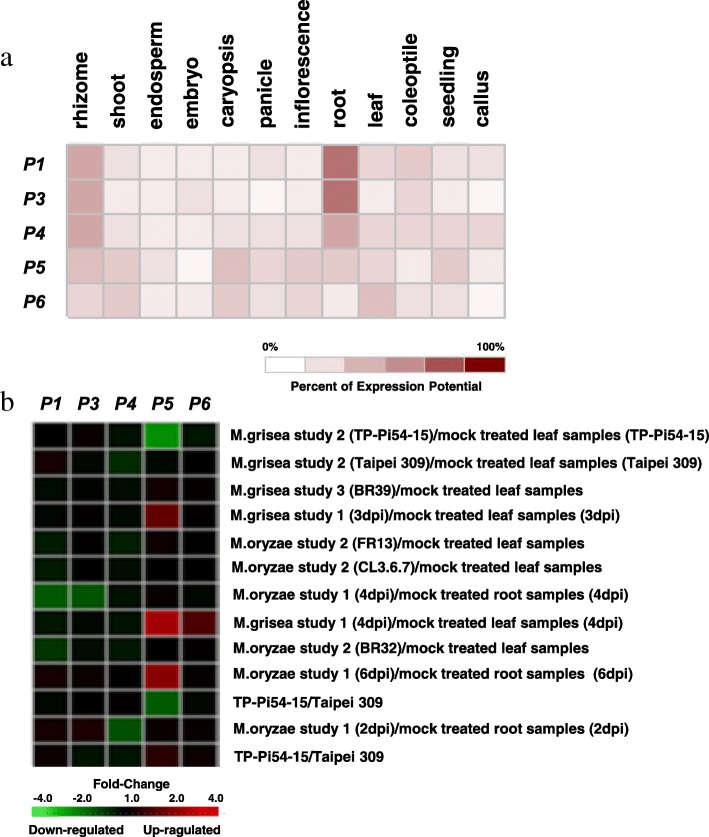
Expression patterns of five candidate genes *P1*, *P3-P6* based on microarray data from the GENEVESTIGATOR. **a** Expression patterns of five candidate genes in various rice tissues; **b** Expression patterns of five candidate genes in the seedling stage in response to *M.oryzae* treatments. Heat map showing the levels of gene expression in different rice tissues and blast inoculated seedlings. The *P2* gene could not be detected in the microarray data

The expression profiles of five candidate genes *P1*, *P3-P6* in various rice tissues and blast fungi inoculated seedlings were investigated based on microarray data deposited in the GENEVESTIGATOR (Fig. 4). The expressions of *P1*, *P4*, *P5* and *P6* could be detected with lower level in panicles, while no expression of *P3* in panicle (Fig. 4a). *P1*, *P3* and *P4* showed similar expression patterns with obviously higher levels in root and rhizome than other tissues. *P5* was expressed in rhizome, shoot, caryopsis, inflorescence, root and seedling with lower level, and *P6* was expressed in shoot, caryopsis and leaf with low level (Fig. 4a).

The expression of *P5* was obviously induced at 3 d, 4 d and 6 d after inoculated with blast fungi, and the expression of *P6* was induced at 4 d after inoculation. However, the expressions of *P1*, *P3* and *P4* were not induced after inoculation of blast fungi (Fig. 4b).

**Fig. 5:**
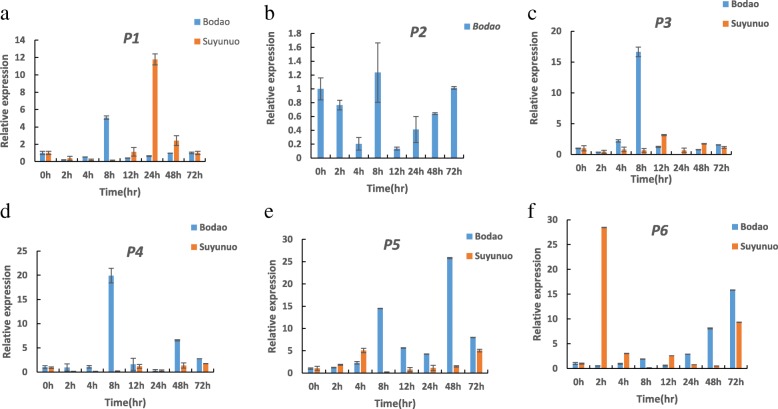
Expression patterns of six candidate genes *P1*-*P6* in Bodao and Suyunuo in immature panicles by real-time PCR methods (**a**-**f**). The *P2* gene could not be amplified from Suyunuo by PCR. The immature panicles were inoculated by *Hoku1* isolate. 18S-rRNA was used as an internal control. Data represent means and standard errors of three replicates.

The expression patterns of six candidate genes in Bodao and Suyunuo were detected in immature panicles inoculated by the blast with real-time PCR approach. The expressions of *P3*, *P4* and *P5* were obviously induced by blast-inoculation in Bodao, while no significantly induction in Suyunuo (Fig. 5). The expressions of *P1* and *P6* were induced in both Bodao and Suyunuo, but with different patterns. *P1* was induced and reached the peak at 8 h in Bodao, while reached the peak at 24 h in Suyunuo (Fig. 5). The expression of *P6* was stably induced and reached the peak at 72 h in Bodao, while it was dramatically induced at 2 h, then decreased to the normal level in Suyunuo (Fig. 5). The expression of *P2* was not prominently induced in Bodao.

### The introgression lines of *Pb-bd1* with enhanced panicle resistance

**Fig. 6:**
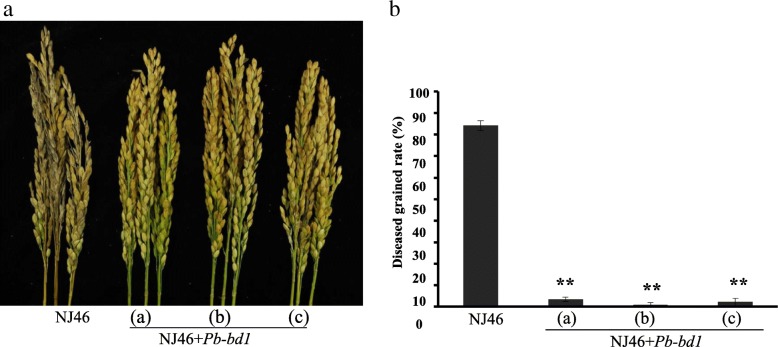
Three introgression lines of NJ46 + *Pb-bd1* with enhanced panicle blast resistance. **a** The resistance phenotypes of NJ46 and the three introgression lines NJ46 + *Pb-bd1* (**a**-**c**). **b** The diseased grain rates of NJ46 and the three introgression lines NJ46 + *Pb-bd1* (**a**-**c**)

Three *Pb-bd1* BC_3_F_4_ introgression lines NJ46 + *Pb-bd1*(a), NJ46 + *Pb-bd1*(b), NJ46 + *Pb-bd1*(c) were selected by MAS method with the markers RM7654 and BS79 closely linked to *Pb-bd1*. After inoculated with blast strain Hoku1, the rates of diseased grains for three introgression lines and susceptible backcross parent NJ46 were 3.56 ± 1.04%, 0.96 ± 0.96%, 2.35 ± 1.68% and 84.31 ± 2.27%, respectively (Fig. 6). It indicates that *Pb-bd1* could significantly improve the panicle blast resistance of NJ46. Three introgression lines were also grown in natural disease nurseries in Changsha, Hunan province and in Jintan and Ganyu, Jiangsu province in 2016 and 2017, and they also showed enhanced panicle blast resistance (Table 5).

**Table 5:** Panicle blast resistance score of three introgression lines in the natural disease nurseries

Plant materials	2016			2017	
	Changsha, Hunan	Jintan, Jiangsu	Ganyu, Jiangsu	Changsha, Hunan	Jintan, Jiangsu
NJ46	5.0	3.5	3.0	5.0	3.0
NJ46 + *Pb-bd1*(a)	3.0	3.0	1.0	3.5	3.0
NJ46 + *Pb-bd1*(b)	2.5	1.5	1.0	3.0	2.5
NJ46 + *Pb-bd1*(c)	1.0	1.0	1.0	3.0	1.5

The panicle blast score was 1–9 as describe as Ahn (1994)

The three introgression lines and backcross parent NJ46 were grown in the field in Nanjing to identify their agronomic characters. There were no significant differences in grain number per panicle between introgression lines and NJ46. The height of NJ46 + *Pb-bd1*(b) and NJ46 + *Pb-bd1*(c) were 80.33 ± 5.91 cm and 80.33 ± 5.25 cm respectively, significantly shorter than that of NJ46 (96.60 ± 5.28 cm). The 1000-grain weight of NJ46 + *Pb-bd1*(b) and NJ46 + *Pb-bd1*(c) were 29.97 ± 1. 31 g and 30.23 ± 0. 82 g respectively, significantly heavier than that of NJ46 (27.76 ± 1. 03 g). There were no significant differences in plant height and 1000-grain weight between NJ46 + *Pb-bd1*(a) and NJ46 (Table 6, Fig. 6).

**Table 6:** Agronomic characters of three introgression lines and their recurrent parent NJ46

Lines	Plant height (cm)	Grain number/panicle	1000-grain weight (g)	Diseased grain(%)
NJ46	96.60 ± 5.28	142.92 ± 29.08	27.76 ± 1.03	84.31 ± 2.27
NJ46 + *Pb-bd1*(a)	86.33 ± 1.70	138.75 ± 17.98	27.41 ± 0.79	3.56 ± 1.04**
NJ46 + *Pb-bd1*(b)	80.33 ± 5.91*	155.00 ± 14.56	29.97 ± 1.31**	0.96 ± 0.96**
NJ46 + *Pb-bd1*(c)	80.33 ± 5.25*	146.50 ± 32.85	30.23 ± 0.82**	2.35 ± 1.68**

** means *P* ≤ 0.01, and * means *P* ≤ 0.05

## Discussion

### The panicle blast resistant phenotypes in RILs population were relatively stable in various trials

Bodao, one *Japonica* landrace from Taihu lake region, exhibited high leaf blast resistance (Li et al. 2007a, 2007b; Huan et al. 2014). In this study, Bodao showed high panicle blast resistance with less than 30% the diseased grains, while the diseased grain rate of Suyunuo was over 60–100% in three various trials. The resistance to panicle blast of each RIL was a significant correlation in various trails (*P* ≤ 0.01). It indicates that the panicle blast resistant phenotypes in RILs population were relatively stable in various trials.

### Two panicle blast resistance QTLs *qPb11–1*(*Pb-bd1*) and *qPb6–1* from Bodao were detected by inoculated with Hoku1

Two panicle blast resistance QTLs *qPb11–1*(*Pb-bd1*) and *qPb6–1* were detected from Bodao and Suyunuo by inoculated with the strain Hoku1 in this study. The *qPb11–1* (*Pb-bd1*) was the major QTL for panicle blast resistance on chromosome 11 and with 71.68% of contribution to resistance phenotype. Up to date, only one panicle blast resistance gene *Pb1* has been cloned and three QTLs conferring panicle blast resistance *qPbm11, Pb-jnw1* and *qPbh-11–1* were identified on chromosome 11 (Hayashi et al. 2010; Ishihara et al. 2014; Wang et al. 2016; Fang et al. 2016). *Pb1*, encoding an atypical CC-NBS-LRR protein, was located on the region between M35 and M26 on the short arm of chromosome 11 (Hayashi et al. 2010). The *qPbm11* was a major panicle blast resistance QTL flanked by SNP markers aa11000537 and aa11001573 identified in *japonica* cultivar Miyazakimochi (Ishihara et al. 2014). The *Pb-jnw1* flanked by SSR markers RM27273 and RM27381 was a major QTL in Jiangnanwan, conferring resistance to both seedling blast and panicle blast (Wang et al. 2016). The *qPbh-11–1* flanked by SSR markers RM27187 and RM27381 was a major QTL for panicle blast resistance in Heikezijing (Fang et al. 2016). The *qPb11–1* (*Pb-bd1*) identified in this study was not located in the same region of *Pb1* and *qPbm11*, but within the region of QTLs *Pb-jnw1* and *qPbh-11-1* (Additional file 3: Figure S1). *qPb11–1* (*Pb-bd1*), *Pb-jnw1* and *qPbh-11-1* are identified from landraces Bodao, Jiangnanwan and Heikezijing, respectively, which are all from Taihu lake region. These three QTLs might be the same novel panicle blast resistance gene and it could be confirmed through fine mapping and cloning methods.

One minor panicle blast resistant QTL *qPb6–1* was detected between RM3431 and RM19951 on chromosome 6 in Suyunuo, and explained 3.54–6.98% of phenotype variance. In our previous results, a minor panicle blast resistant QTL *qPbj-6-1* was also detected on chromosome 6 in Suyunuo with the Jiangnanwan×Suyunuo F_2:6_ RIL population (Wang et al. 2016). Both of *qPb6–1* and *qPbj-6-1* were located in the *Pi2/Pi9* cluster (Wang et al. 2016). *qPb6–1* was detected in two trails in Lingshui in 2014 and in Nanjing in 2015 in this study, while *qPbj-6-1* was detected only in one trial (Wang et al. 2016). It indicates that these minor QTLs might be greatly influenced by environmental factors in the fields.

### Six candidate genes in *Pb-bd1* region were predicted

With the fine mapping populations, *Pb-bd1* was finally mapped in a region of 40.6 kb between markers BS83 and BS98, and there were six candidate genes predicted. Among these six candidate genes, *P1* encodes a NAC domain-containing protein, and *P5* and *P6* encode VWA containing proteins. NAC domain containing protein and VWA-containing domain protein have been reported that they can play important roles in rice- blast interactions (Liu and Jambunathan 2005; Lin et al. 2007; Rawat et al. 2012; Sun et al. 2013). *P2* encoding unknown expression protein was only existed in resistant cultivar Bodao, and it could be the possible resistance gene *Pb-bd1*. *P3* and *P4* encode NBS-LRR type disease resistance proteins. Among the cloned 28 resistant genes, 24 genes encode NBS-LRR-containing proteins, and six resistance genes as *Pikm*, *Pia*, *Pikp*, *Pike*, *Pi-l* and *Pi-5* were contributed by two adjacent NBS-LRR resistance genes (Zhai et al. 2011; Hua et al. 2012; Chen et al. 2015; Yuan et al. 2011; Ashikawa et al. 2008). These adjacent candidate NBS-LRR genes *P3* and *P4* could also be the resistance gene *Pb-bd1*. In our future research, we will further validate the functions of these six candidate genes through gene editing or transgenic complementary methods.

### Introgression lines with high panicle blast resistance were good resources for blast resistance breeding

It has been proved that the most effective method to control rice blast disease is using resistance genes (Hulbert et al. 2001). Marker-assisted selection (MAS) is a high effective strategy to introduce the resistance genes into susceptible commercial cultivars. It has been reported that 99.75% of the plants containing the target gene were selected within 5 cM genetic distance between the marker and target gene by the MAS method (Zheng et al. 2009). In this study, three *Pb-bd1* introgression lines were developed by MAS method. Compared with NJ46, the *Pb-bd1* introgression lines enhanced resistance to panicle blast over 80%, while the QTL *qPb11–1* (*Pb-bd1*) could only explain 55.31–71.68% of resistance phenotypic variance in Bodao. The possible reason could be due to some resistant locus in NJ46 might function with *Pb-bd1* to contribute the resistance in the three introgression lines. The agronomic characters of introgression lines showed less significant differences with NJ46. Therefore, Bodao and the introgression lines with high panicle blast resistance were good resources for application in blast resistance breeding.

## Additional files

Additional file 1: **Table S1.** Primers of PCR-based markers used for fine mapping *Pb-bd1*. (DOCX 17 kb)

Additional file 2: **Table S2.** Primers for expression patterns analysis of candidate genes by real-time PCR. (DOCX 16 kb)

Additional file 3: **Figure S1.** The integrated physical map of four panicle blast resistance QTLs. (PPTX 79 kb)

## Abbreviations

MAS: Molecular assisted selection
NBS-LRR: Nucleotide binding site-leucine rich repeat
NJ46: Nanjing46
PAGE: Polyacrylamide gel electrophoresis
QTLs: Two quantitative trait loci
RGP: Rice Genome research Program
VWA: Von Willebrand factor type A domain
