# Supplementary material for: Fine mapping of a panicle blast resistance gene Pb-bd1 in Japonica landrace Bodao and its application in rice breeding
Source: Rice (N Y). 2019 Mar 25;12:18. doi: 10.1186/s12284-019-0275-0 (PMC6434012; doi:10.1186/s12284-019-0275-0)
Supplement: Supplementary file 1 — Table S1. Primers of PCR-based markers used for fine mapping Pb-bd1. (DOCX 17 kb) [file 12284_2019_275_MOESM1_ESM.docx]

Supplementary Table S1 Primers of PCR-based markers used for fine mapping *Pb-bd1*

| Marker  name | Markertype | Forward primer (5'-3') | Reverse primer(5'-3') |
| --- | --- | --- | --- |
| BS23 | InDel | CGCGACTAATCTAGACTCT | TGGACACTTATGCAATTAGC |
| BS59 | InDel | CGCAGTAACCCAGATA | AGTCAACGGTGTCAAAT |
| BS83 | InDel | GAAATCTCCGAGGACGAA | GCTGCCGCCTCTATCT |
| BS84 | InDel | CTCTGGCTGTCGGAATG | ACCCGATCTTGTTCAACTTAT |
| BS86 | InDel | CCCAGCCCAACTCCCTA | GCAGCGTCAGCCAAAA |
| BS90 | InDel | TCGCCTTTGCCCGAGAC | TGCCGATTCAATTAGGTACTTTCT |
| BS97 | InDel | GAGCATGTAAGCATTTTGAT | AGCCCTTAGTTTAGCAATCA |
| BS98 | InDel | GTGGCGATCACGGCGGAGAA | CGGCGTTGAAACGGTAGCTG |
| RM7654 | SSR | CGCAGCTCCTCCTAATAAACTGTGG | TTACCTAAGCAGCTGGCACTGATGG |
