# Supplementary material for: Fine mapping of a panicle blast resistance gene Pb-bd1 in Japonica landrace Bodao and its application in rice breeding
Source: Rice (N Y). 2019 Mar 25;12:18. doi: 10.1186/s12284-019-0275-0 (PMC6434012; doi:10.1186/s12284-019-0275-0)
Supplement: Supplementary file 2 — Table S2. Primers for expression patterns analysis of candidate genes by real-time PCR. (DOCX 16 kb) [file 12284_2019_275_MOESM2_ESM.docx]

Supplementary Table S2 Primers for expression patterns analysis of candidate genes by real-time PCR

| Gene | Forward primer (5'-3') | Reverse primer (5'-3') | Melting temperature(℃) |
| --- | --- | --- | --- |
| *P1* | TGGGGAGCCGTGGTTCTACTTC | TGCGGTCTTCCAATACCCTGA | 55 |
| *P2* | GCCATCCACCGCTGCTCG | GGTGACAAGCCTGAGTGGTG | 56 |
| *P3* | CCTCATGTCGGTATCAACG | CGTGGTCTTGCCTATTCC | 56 |
| *P4* | CCAAGACTCATCTCCAATGC | GACTGTATCACTTGCATCCC | 57 |
| *P5* | CCATCTTCCCCACAATCCCAA | CTGACATCAAGCACCGCGACTA | 56 |
| *P6* | CACGAGCTCAGACGAACAAG | GACACGACCCTTGAGATCCA | 55 |
| *18S* | GAGACATTCAGCGTTCCAGC | GCATAACCTTCGTAGATTGGGAC | 58 |
