# Supplementary material for: Fine mapping of a panicle blast resistance gene Pb-bd1 in Japonica landrace Bodao and its application in rice breeding
Source: Rice (N Y). 2019 Mar 25;12:18. doi: 10.1186/s12284-019-0275-0 (PMC6434012; doi:10.1186/s12284-019-0275-0)
Supplement: Supplementary file 3 — Figure S1. The integrated physical map of four panicle blast resistance QTLs. (PPTX 79 kb) [file 12284_2019_275_MOESM3_ESM.pptx]

## Slide 1
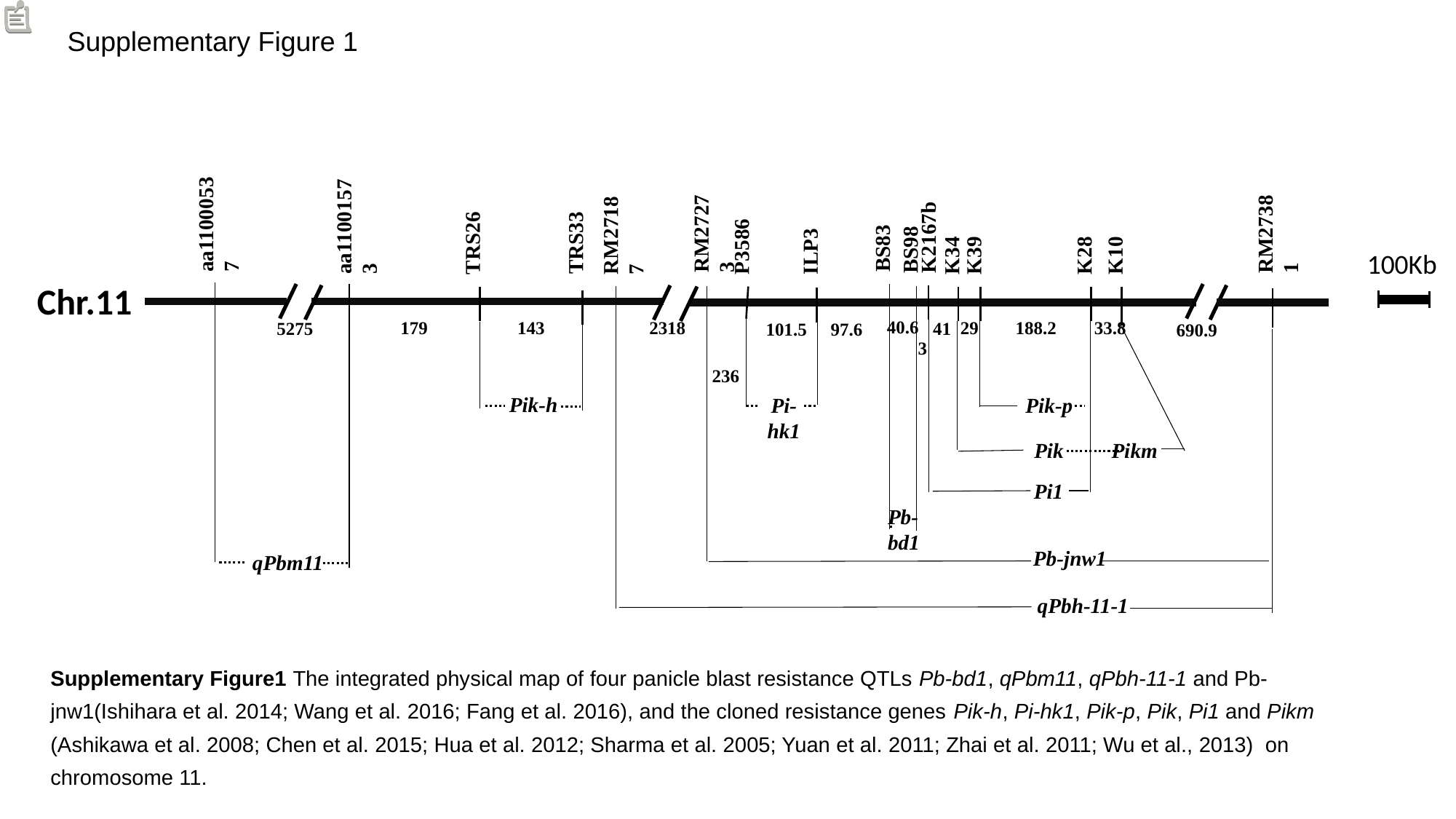

Supplementary Figure 1
aa11000537
aa11001573
BS83
RM27273
K2167b
TRS33
TRS26
Pik-h
P3586
ILP3
Pi-hk1
K28
Pi1
K34
Pik
K39
Pik-p
K10
Pikm
2318
188.2
143
33.8
101.5
3
29
RM27381
RM27187
97.6
690.9
Pb-jnw1
qPbh-11-1
Chr.11
40.6
179
5275
Pb-bd1
qPbm11
BS98
100Kb
236
41
Supplementary Figure1 The integrated physical map of four panicle blast resistance QTLs Pb-bd1, qPbm11, qPbh-11-1 and Pb-jnw1(Ishihara et al. 2014; Wang et al. 2016; Fang et al. 2016), and the cloned resistance genes Pik-h, Pi-hk1, Pik-p, Pik, Pi1 and Pikm (Ashikawa et al. 2008; Chen et al. 2015; Hua et al. 2012; Sharma et al. 2005; Yuan et al. 2011; Zhai et al. 2011; Wu et al., 2013) on chromosome 11.
